# Genome language modeling (GLM): a beginner’s cheat sheet

**DOI:** 10.1093/biomethods/bpaf022

**Published:** 2025-03-25

**Authors:** Navya Tyagi, Naima Vahab, Sonika Tyagi

**Affiliations:** AI and Data Science, Indian Institute of Technology, Madras, Chennai 600036, Tamil Nadu, India; Amity Institute of Integrative Health Sciences, Amity University, Gurugram 122412, Haryana, India; School of Computing Technologies, Royal Melbourne Institute of Technology (RMIT) University, 3001 Melbourne, Australia; School of Computing Technologies, Royal Melbourne Institute of Technology (RMIT) University, 3001 Melbourne, Australia

**Keywords:** natural language processing, genomics, digital health, precision medicine, machine learning, AI

## Abstract

Integrating genomics with diverse data modalities has the potential to revolutionize personalized medicine. However, this integration poses significant challenges due to the fundamental differences in data types and structures. The vast size of the genome necessitates transformation into a condensed representation containing key biomarkers and relevant features to ensure interoperability with other modalities. This commentary explores both conventional and state-of-the-art approaches to genome language modeling (GLM), with a focus on representing and extracting meaningful features from genomic sequences. We focus on the latest trends of applying language modeling techniques on genomics sequence data, treating it as a text modality. Effective feature extraction is essential in enabling machine learning models to effectively analyze large genomic datasets, particularly within multimodal frameworks. We first provide a step-by-step guide to various genomic sequence preprocessing and tokenization techniques. Then we explore feature extraction methods for the transformation of tokens using frequency, embedding, and neural network-based approaches. In the end, we discuss machine learning (ML) applications in genomics, focusing on classification, regression, language processing algorithms, and multimodal integration. Additionally, we explore the role of GLM in functional annotation, emphasizing how advanced ML models, such as Bidirectional encoder representations from transformers, enhance the interpretation of genomic data. To the best of our knowledge, we compile the first end-to-end analytic guide to convert complex genomic data into biologically interpretable information using GLM, thereby facilitating the development of novel data-driven hypotheses.

## Introduction

Natural language processing (NLP) is a sub-field of computer science and artificial intelligence (AI) focused on the interaction between computers and human languages. It allows computers to understand, interpret, and generate human language in a way that is meaningful to machines. NLP applications have evolved significantly over time, starting with foundational tasks and progressing to advanced technologies. Early applications included text classification, where algorithms categorized text into predefined labels, and machine translation, which converted text from one language to another. Sentiment analysis followed, enabling the extraction of emotional tone from text, and speech recognition, which transformed spoken language into written text. More advanced applications like named entity recognition (NER) and information retrieval emerged, focusing on extracting relevant entities and data from large text corpora. Language modeling techniques such as next sentence prediction (NSP), masked language modeling (MLM), and the development of large language models (LLMs) represent the latest advancements in NLP. These models are proving crucial for understanding, generating, and predicting natural language, and a wider use of such applications is gaining momentum.

Genomic information consists of DNA sequences encoding the genetic code and translating it into functional biomolecules such as RNA or protein. This information can be represented using four nucleotide alphabets (also known as bases) namely, A, T, G, and C as unstructured text. In recent years, NLP has found several applications in the field of genomics involving analyses using DNA/RNA, or protein sequences, all of which can be represented as text. For example, NLP-based algorithms were used for protein sequence classification [1], for identifying DNA modification sites [2], functional annotations of co-expressed gene pairs [3], prediction of gene promoters [4], RNA modifications sites [5], enhancers [6], DNA replication origins [7], for generating pseudo nucleotide composition [8] or even representation of protein sequences [9].

The use of NLP techniques for genome language modeling (GLM) is appealing because genomic sequences can be processed in a manner similar to natural language text sequences. Therefore, GLM involves using NLP models to interpret and predict genetic sequences, treating them as a “language” with its own syntax and semantics. This approach enables the development of models that can identify and predict genetic features from sequence data alone, enhancing our understanding of biological grammar. However, the grammar it follows and the distinctions of genomic “words” are not as apparent. Despite these challenges, this innovative approach enables the development of models that can identify and predict genetic and genomic features and enhance our understanding of biological grammar by using sequence data alone.

The field of genomics and genetics has seen a rapid growth in the application of machine learning over the past two decades, which has been extensively reviewed previously [10–12]. This also comes with several challenges and pitfalls [13]. GLM represents a significant advancement in computational biology by applying NLP techniques to uncover insights from complex biological data. By applying GLM, researchers can develop more accurate predictive models, improve functional annotations, and gain a deeper understanding of the underlying mechanisms of gene expression and regulation. This commentary explores the current opportunities and challenges in the field of GLM, providing an overview of its methodologies and demonstrating its typical workflows. We specifically review machine learning (ML) approaches that treat and process genomic data as a text modality where language modeling techniques from the computational linguistics apply. We will cite specific applications of these methods for biological sequences showing their potential in tasks such as gene and protein sequence prediction, and interpreting biomedical and clinical text data. Where possible, we will provide a comparative analysis of algorithms. While we will use DNA sequences as our example data to illustrate different methodologies, these same methods can also be applied to other types of biological sequence data, such as RNA or protein sequences.

## GLM workflow of genomic sequence analysis

**Figure 1.**
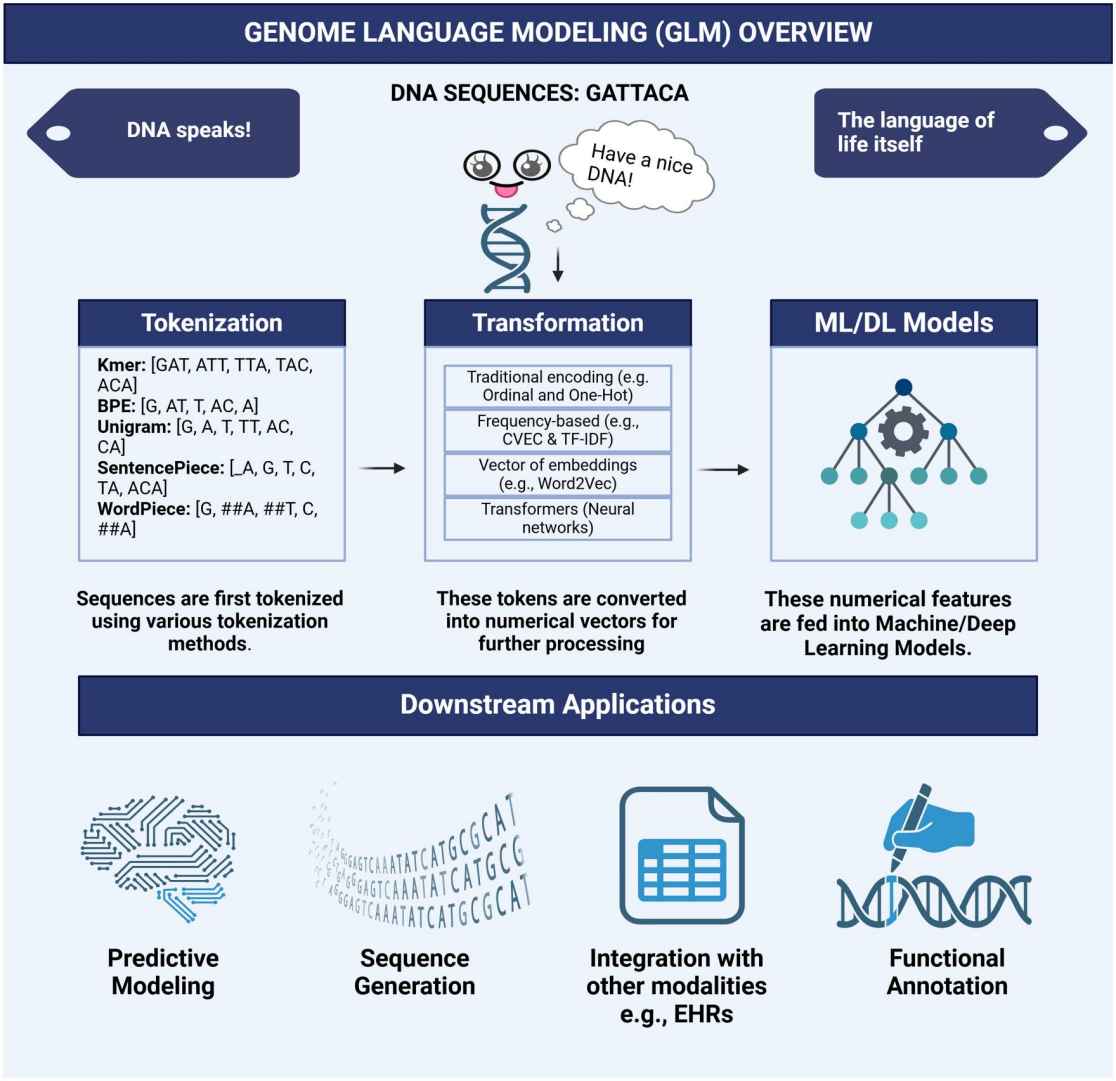
Decoding the language of life: GLM Overview: a graphical abstract outlining the GLM approach, detailing the steps from DNA sequence tokenization, numerical transformation, and application of ML/DL models for downstream tasks like predictive modeling and functional annotation.

In this section, we provide an overview of a typical workflow of GLM (Fig. 1) that involves genomic sequence analysis using ML techniques. The workflow includes steps such as genomics sequence representation, feature extraction methods, ML applications including functional annotation. These methods are crucial for understanding how raw genomic data can be transformed into meaningful insights through NLP methods.

### Feature extraction

Feature extraction is a vital step in the analysis and modeling of genomic sequences that involves the preprocessing and tokenizing steps.

#### Preprocessing

Before numerical encoding, biological sequence data often undergoes preprocessing steps to enhance quality and usability. Common preprocessing techniques include handling length, removing unwanted characters, and normalization.

**Figure 2.**
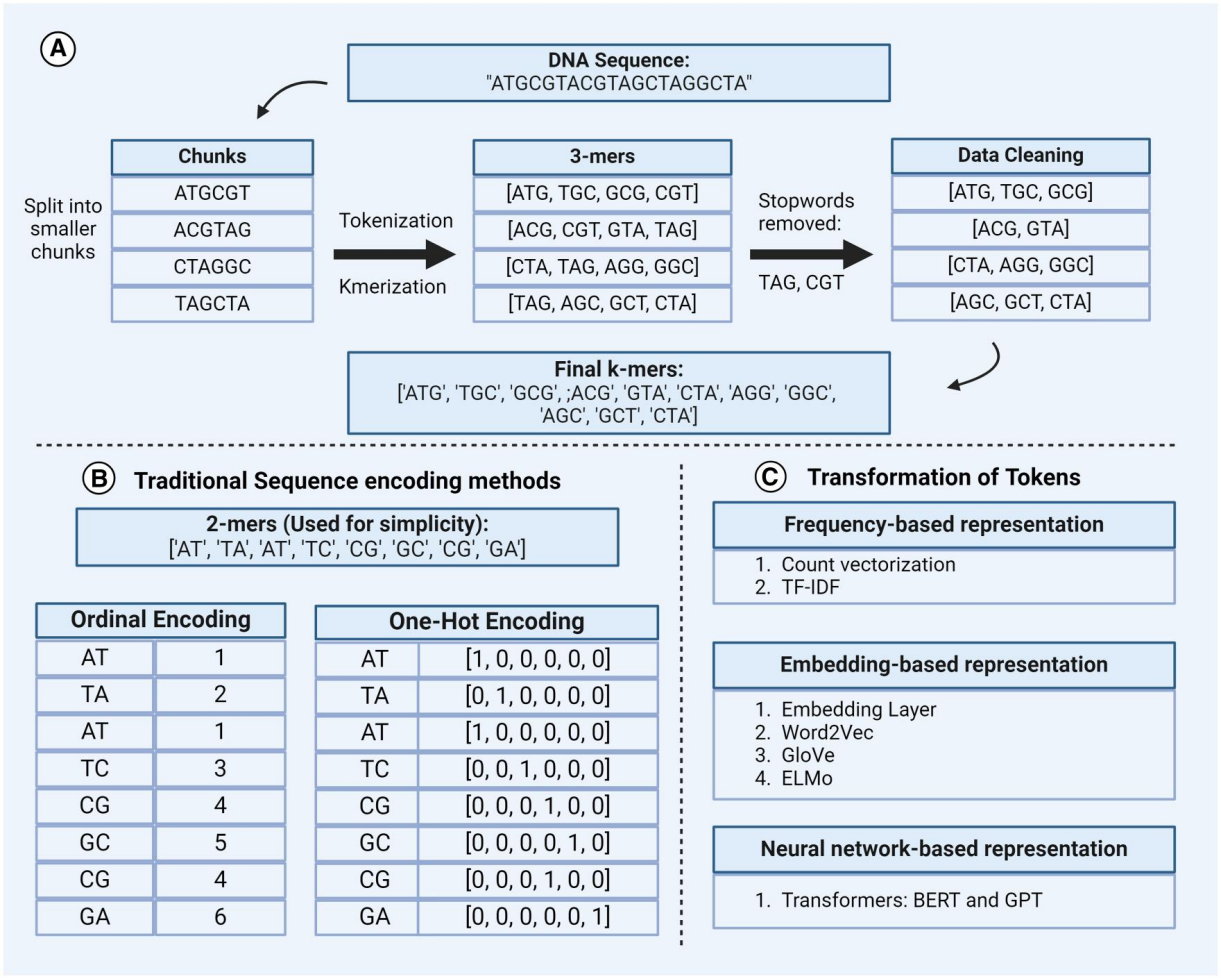
A GLM Workflow cheat sheet illustrating the following: (**A**) The complete process of fragmenting long DNA sequences into smaller chunks, tokenizing them as 3-mers, and then performing stopword removal. (**B**) Traditional methods for encoding or transforming DNA sequences, including ordinal and one-hot encoding. (**C**) Advanced approaches to token transformation using frequency-based representation, embedding, and neutral network representation.

Due to the extensive length of DNA sequences, which can range from hundreds to hundreds of millions of base pairs (bp), preprocessing involves segmenting these sequences into smaller, manageable chunks. This step is essential to reduce computational costs and enable downstream algorithms to efficiently process and analyze relevant sub-sequences (Fig. 2A). Filtering out common genomic terms or sequences containing little or unusable information, such as the character “N” in nucleotide sequence data (Fig. 2A) and normalizing sequences to a uniform format is performed, addressing variations like case sensitivity or character encoding.

#### Tokenization

**Figure 3.**
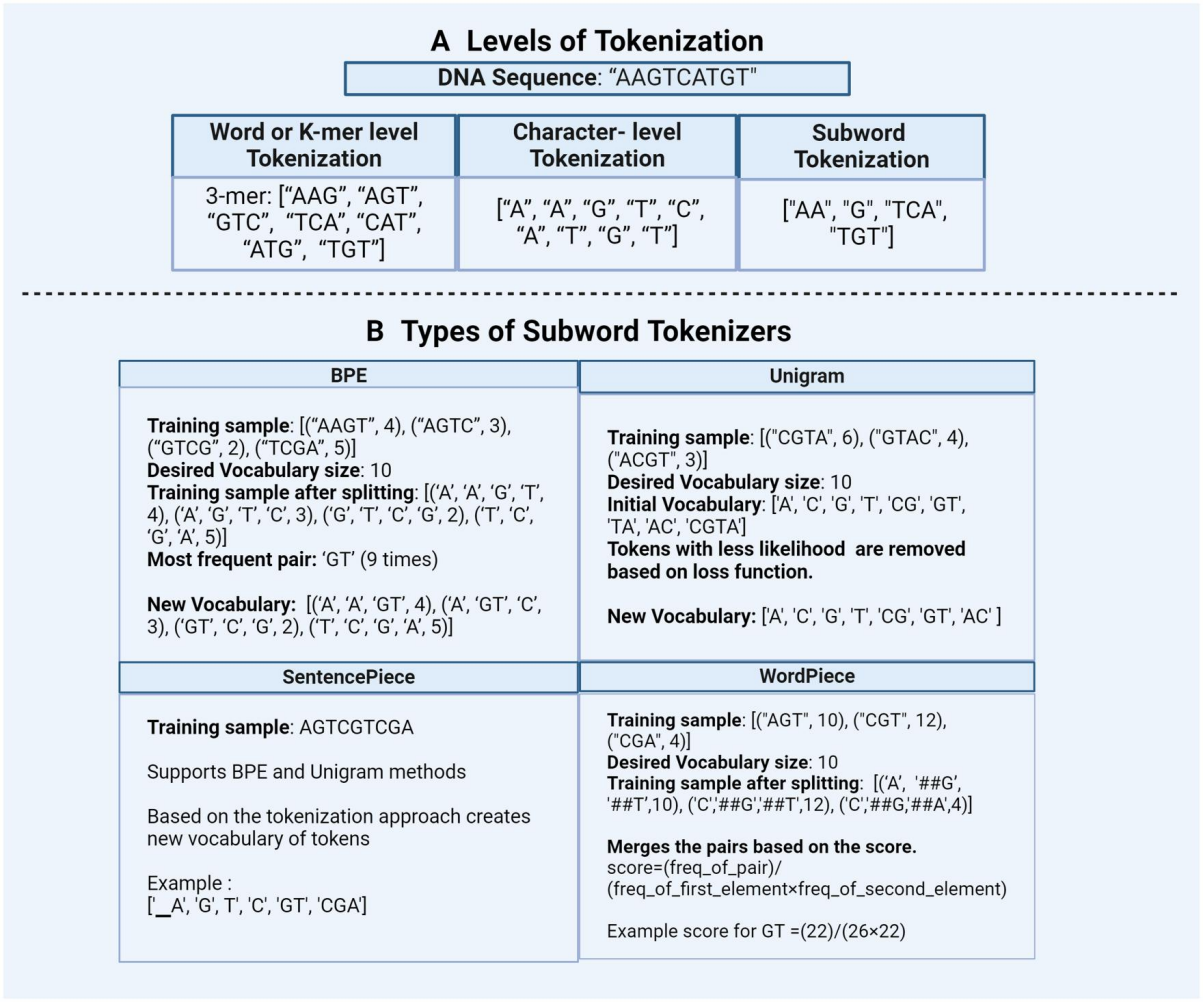
This figure illustrates examples of: (**A**) Level of Tokenization: The section illustrates the various levels of tokenization applied to a DNA sequence input, producing different tokenized outputs. (**B**) Types of Subword Tokenizers: The subword tokenization techniques like BPE, Unigram, WordPiece, and SentencePiece are demonstrated using a DNA sequence (AAGTCATGT) example. Various steps of each tokenization leading to the final vocabulary are listed.

In the preprocessing step, tokenization involves breaking down long DNA, RNA, or protein sequences into smaller units called “tokens.” These tokens serve as the basic building blocks for subsequent analysis (Fig. 3A). These are equivalent to “words” of human language. These tokens can be either reversible or non-reversible, depending on whether they can be converted back to their original sequence. Irreversible tokens are often used as a security measure, particularly when data is shared for third-party analytics or in less secure environments. Tokenization can be performed in multiple different ways using pre-defined rules for the length and frequency of tokens or following data-driven approaches. Table 1 gives a summarized overview of different types of tokenization. Furthermore, various sequence processing methods are summarized in Supplementary Table 1.

**Table 1.** Overview of tokenization types.

Tokenization approach	Method	Pros	Cons	Reversibility
**Character tokenization**	Splits the data into a set of characters.	Handles OOV words by breaking them into characters. Also, it has a limited size of vocabulary since it contains only a unique set of characters.	It does not capture the semantic relationship between the tokens.	Non-reversible
**Word tokenization**	Splits the data into tokens using a certain delimiter.	Large amounts of text can be easily tokenized without using complex computation.	Fails at handling OOV words. Furthermore, does not scale well with big datasets as it generates a huge amount of vocabulary.	Reversible
**K-mer based tokenization**	The data is split into fragments of desired k-length.	Computationally less expensive method to generate tokens for genomic sequences.	Does not capture the relationship between the tokens and generates a larger vocabulary.	Non-reversible
**Subword tokenization**	Splits the data into subwords (or n-gram characters). The most frequently used tokens are given unique IDs and the less frequent tokens are split into subwords.	Transformer-based models use this algorithm for preparing vocabulary. Has a decent vocabulary size. It does capture the semantic relationship between the tokens.	Increases computational cost while reducing the model interpretability.	Non-reversible

##### Word or K-mer level

In the context of biological sequences, k-mer is a sub-sequence of k length (e.g. 3-mers and 4-mers). K-mers are usually generated by shifting an overlapping window of size k throughout the sequence (Fig. 3A). Large amounts of data can be easily tokenized by this method. Since we generate all overlapping k-mers of a given length, the resulting number of tokens is very high, leading to a high computational cost. A number of initial methods have taken an ad-hoc approach to determine the values of “k” but lately more comprehensive computational approaches have been discussed to strike a tradeoff between computational complexity and biological signal [14]. Another drawback of this type of tokenization method is that they do not accommodate out-of-vocabulary (OOV) words. OOV words are words that are absent from the vocabulary of a language model or tokenizer. These words may include misspellings, rare words, or domain-specific terms not encountered during model training. Additionally, this approach fails to capture the semantic relationship between the tokens, limiting its effectiveness in understanding the context and meaning of sequences. Various GLM works have used k-mer tokenization to break down considerably large genomic sequences for subsequent analysis [4, 15–18].

##### Character level

This tokenization method splits the sequences into individual characters, effectively handling OOV words by breaking them into their component characters. This results in a significantly smaller vocabulary size, as it includes only the four nucleotide characters (A, T, G, C). However, this approach loses semantic relationships between tokens, as the context and meaning derived from longer sequences are not captured. Additionally, while the vocabulary size is reduced compared to the word or k-mer level tokenization, the overall length of the tokenized sequence increases, making the process non-reversible and less efficient for capturing higher-level features (Fig. 3A). Transformer-based models like RNABERT [19] tackle these limitations by combining character-level tokenization with position embeddings to capture long-range dependencies within RNA sequences. T However, traditional transformer-based models only use a fixed amount of tokens which gives them a disadvantage when dealing with long-range sequences. HyenaDNA [20] and Evo [21] that are based on StripedHyena architecture also use character-level tokenization to capture information at a single nucleotide level and long-range dependencies within genomic sequences. These models are employed in various tasks such as predicting gene regulatory elements and protein structure prediction.

##### Subword Tokenization

Subword tokenization divides sequences into smaller units or subwords. N-grams, sequences of N tokens, are commonly used [22, 23]. The most frequent words receive unique IDs, while the less frequent words are split into subwords. This approach addresses the drawbacks of word and character-level tokenization, such as large vocabulary sizes, inability to handle OOV tokens, and increased sequence lengths, by breaking them into known subwords. It also maintains the semantic relationships between tokens. Typically, subword tokenizers use pre-tokenized input rather than raw data (Fig. 3A and B). Table 2 presents a concise overview of the subword tokenization types.

**Table 2.** Overview of sub-word tokenization techniques.

Method name	Type of model	Feature
**Byte pair encoding (BPE)**	Frequency-based	Initially developed as a compression algorithm, found applicability in sub-word tokenization using frequency-based merge rules.
**WordPiece**	Score-based	Select tokens based on a scoring mechanism to create an effective tokenizer model.
**Unigram**	Probability and loss function based	By quantifying a loss function, iteratively removes less efficient tokens from a larger vocabulary based on their probabilities to build a fixed size vocabulary.
**SentencePiece**	Allows both BPE and Unigram tokenization	Does not require pre-tokenization and is language independent. Provides flexible integration with BPE, Wordpiece, and Unigram methods

###### Byte pair encoding

Byte pair encoding (BPE) is a compression algorithm, that is, it encodes or compresses data and is widely used in NLP pipelines to perform subword tokenization [24]. It addresses the drawbacks of word- and character-level tokenization as it handles OOV tokens and has a limited vocabulary size. Vocabulary size of BPE can be a user-defined parameter. Initially, the vocabulary consists of unique characters or subwords of various lengths from the tokens in the training sample along with their respective frequencies. The tokens in the training sample are first split into individual characters from the vocabulary. These characters represent the smallest units of the words. The algorithm then identifies the most frequent combinations of adjacent characters, sub-words, or multi-character sequences and merges them to form new subwords. This process is repeated iteratively, with the most frequent sequences being merged at each step, gradually building up more complex subwords until the entire vocabulary is either compressed to the desired size or fully merged (Fig. 3B). However, since BPE implements a simple merging rule based on the most frequent tokens, it does not account for the semantics of the tokens which can result in non-meaningful subwords. This leads to sub-optimal tokenization. BPE is employed in language models like GPT-2 [25], XLM [26], and FlauBERT [27]. K-mer tokenization introduces computation and sample inefficiencies which is a significant challenge in developing large genomic models. GenaLM [28] and DNABERT-2 [29] are the genomic foundational model that replaces k-mer with BPE to tackle these cons of k-mer tokenization. BPE has been used to encode protein sequences to increase the efficiency of models like seq2seq [30].

###### Unigram

The unigram [31] model solves the merging problem of BPE by calculating the likelihood of each subword combination rather than picking the most frequent pattern. Initial vocabulary of unigram consists of different combinations of tokens created by the frequency or BPE-based approach. Subword tokens with the highest losses are removed from this vocabulary at each iteration step until the desired vocabulary size is achieved (Fig. 3B). This ensures that the model retains subwords that are not only frequent but also meaningful. The unigram approach is usually used in conjunction with SentencePiece in popular architectures such as BigBird [18] and XLNet [32]. Protein sequence tokenization often faces a challenge with representing the residues along with the amino acids. GLM models use unigram to create advanced residue vocabularies instead of seeing them as isolated tokens [33]. This approach has been successfully implemented in another work by us [34].

###### Specialized subword tokenizer

- SentencePiece: SentencePiece is a modified version of subword units approach [35]. It is an effective and language-independent subword tokenizer, owing to its pre-tokenization-free approach. During tokenization, SentencePiece treats the sentences as raw texts and defines a fixed vocabulary size for creating the vocabulary. It then converts all characters into Unicode including whitespaces. This feature helps to handle accurate reverse conversion from detokenized tokens to original ones (lossless tokenization). This feature makes it an effective approach for biological sequences as well. SentencePiece also gives flexibility to choose between BPE and Unigram as subword algorithms which improves the robustness of the entire tokenization approach (Fig. 3B). Transformer models like ALBERT [36], XLNet [32], and T5 [37] use SentencePiece in conjunction with Unigram. SentencePiece has been used in bacterial genome tokenization, using entire genomes for strain classification [38]. BioALBERT, a domain-specific model trained on biomedical and clinical data uses Sentencepiece to perform downstream tasks like NER, sentence similarity, relation extraction, and document classification [39].
- WordPiece: WordPiece [40] is a tokenization approach similar to BPE (Fig. 3B). It has similar merge rules like BPE but differs in the selection of token pairs to be merged. WordPiece starts by creating a vocabulary of tokens from the initial words by splitting the word into each character and appending WordPiece prefix “##.” It then merges the tokens based on the below scoring formula. According to this formula, the pairs which appear less frequently in the text get merged than those which appear too frequently. WordPiece was developed by Google to train the bidirectional encoder representations from transformers (BERT) model, then it got reused in many other transformer models like DistilBERT, MobileBERT, and MPNET. In Bio-NLP field, BioBERT [41] and MedBERT [42] use wordpiece tokenizer to perform biomedical text mining and NER tasks.
  (1) $$\text{score}=\frac{\text{freq}\_\text{of}\_\text{pair}}{\text{freq}\_\text{of}\_\text{first}\_\text{element}\;\times \;\text{freq}\_\text{of}\_\text{second}\_\text{element}}$$

### Genomic sequence representation

Representing genomic sequences as features is an essential aspect of GLM. This involves converting DNA sequences, represented by A, C, G, and T characters, (and RNA sequences, in which T is replaced by U character) into numerical forms. Sequence representation involves encoding the tokenized sequences where text sub-units are converted into numerical formats suitable for computational analysis.

#### Sequence encoding techniques

Sequence encoding in GLM has evolved significantly, starting with fundamental encoding techniques such as ordinal encoding [43], where nucleotides are encoded as integers based on their alphabetical order (e.g. A = 1, C = 2, G = 3, T = 4) (Fig. 2B). This method simplifies the representation of sequences into numerical values but may not capture underlying relationships between nucleotides. Another popular encoding method is one-hot encoding, which maps each nucleotide (A, C, G, T) to a binary vector representation (Fig. 2B). In this scheme, each nucleotide is represented as a vector of zeros with a single one indicating its presence (e.g. A = [1, 0, 0, 0], C = [0, 1, 0, 0], etc.). Beyond these foundational methods, other historical encoding techniques include sum, Helmert, polynomial, and binary encoding [43]. These methods aimed to capture more nuanced relationships between categorical values, providing alternatives to simple one-hot or ordinal representations. For instance, sum encoding represents each category by its deviation from the grand mean. In contrast, Helmert’s encoding contrasts each level of a categorical variable with the mean of the subsequent levels. Even though this article focuses more on nucleotide sequences (DNA/RNA), the same approach can be applied to protein sequences that are written using 20 alphabets known as amino acid characters. See Table 3 for a detailed comparison of different encoding algorithms.

**Table 3.** Algorithms comparison.

Aspect	Ordinal Encoding	One-hot encoding	Word embedding	BERT
**Encoding form**	Each token is assigned an integer value.	Each token is represented by a vector (matrix).	Tokens that have the same meaning have similar representations. Each token is represented by real-valued vectors.	BERT provides contextualized embedding by taking into account the context of each token occurrence.
**Semantic relationship**	Does not capture the relationship between tokens.	Does not capture the relationship between tokens.	Captures the relationship between tokens.	It captures the relationship between tokens.
**Categorical variables preferred**	Suitable for ordinal variables but not for nominal.	Suitable for both nominal and ordinal variables.	Suitable for both nominal and ordinal variables.	Suitable for both nominal and ordinal variables.
**Memory consumption and training time**	Lower memory usage but may not scale well with large datasets.	High memory consumption due to large dimensions.	More efficient in terms of memory than one-hot encoding.	High memory consumption but scales with more data for better accuracy.
**Handling OOV (Out of Vocabulary) words**	Cannot handle OOV words.	Cannot handle OOV words.	Struggles with OOV words.	Efficiently handles OOV words with the help of modern tokenizers.

*Note:* This table presents a detailed comparison of different encoding algorithms including ordinal encoding, one-hot encoding, word embedding, and BERT.

#### Transforming tokens using frequency-based representation

Traditional methods of transforming tokens include ordinal encoding, one-hot encoding, and other encoding methods discussed in the earlier section. To further enhance the representation of tokenized sequences, various transformation techniques can be applied. This section explores frequency-based representation methods for the transformation of tokens.

**Figure 4.**
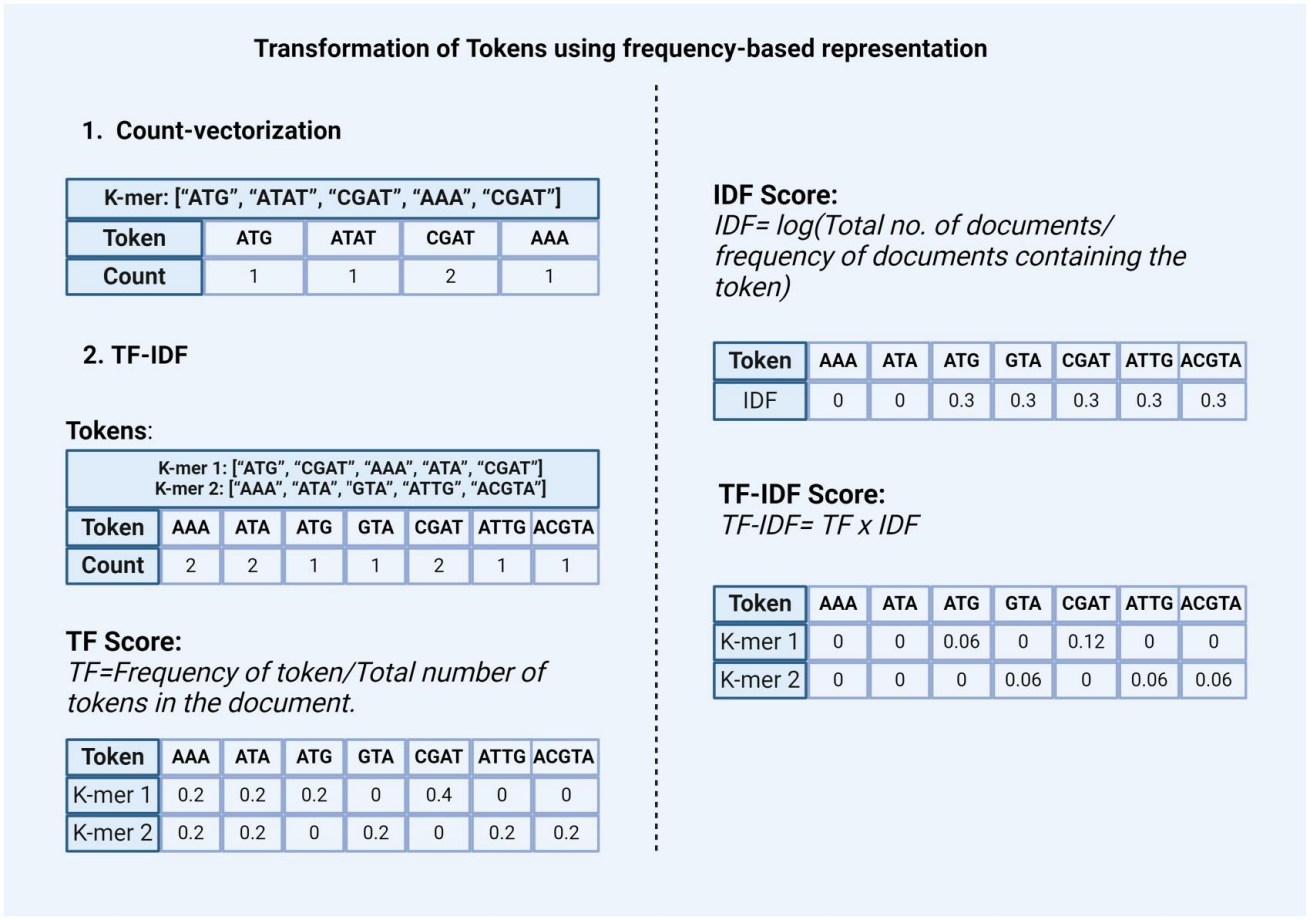
Transformation of DNA sequences using frequency-based representations: (1) Count vectorization and (2) TF-IDF. The example illustrates how DNA sequences are tokenized and then converted into a numerical format.

- Count-vectorization: This method counts the number of occurrences of each token and represents it as a vector or matrix (Fig. 4  (1)). This method considers high-frequency words as more significant. In genomics, countvectorized features combined with Long Short-Term Memory (LSTM) have been used for protein class prediction, outperforming ML algorithms like Naive Bayes [44]. In another study, the count-vectorization method was used for DNA sequence processing [45] and identifying transcription factor-DNA interaction important for gene regulation and expression [46].
- TF-IDF: In the context of natural language data Term Frequency-Inverse Document Frequency (TF-IDF) is a statistic based on the frequency of a token in the document. It is used to analyze the difference between the two documents by the uniqueness of tokens. A higher value of TF-IDF signifies higher importance of the tokens in the corpus while lower values represent lower importance. It is calculated by combining two metrics: Term Frequency (TF) and Inverse Document Frequency (IDF) (Fig. 4 (2)).
- **Term Frequency (TF) score**
  It is based on the frequency of tokens or words in the document. TF is calculated by dividing the number of occurrences of a token (i) by the total number (N) of tokens in the document (j).
  TF (i)=frequency (i, j)/N (j)
- **Inverse Document Frequency (IDF) score**
  It is a measure that indicates how commonly a token is used. It can be calculated by dividing the total number (N) of documents (d) by the number of documents containing the token (i).
  IDF (i)=log(N (d)/frequency (d, i))
  The final TF-IDF score is calculated by multiplying TF and IDF scores.
  TF-IDF (i)=TF (i) × IDF (i)
  Similar to the Count-vectorizer method, TF-IDF also gives priority to the tokens that have the highest frequency of occurrence in the document or the corpus. However, these count-based methods fail to capture the semantic relationship between tokens. TF-IDF has been used for an alignment method to detect laterally originated genomic regions [47] and also for classification purposes in biomedical text mining [48].

#### Transforming tokens using embedding and neural network-based representations

More recently, embeddings and neural network-based representations have been used for token transformations. Embedding is a learned transformation for corpus where we compare tokens by comparing features to find relationships between them. Each token is mapped to one vector. Similar tokens have a representation identical to vectors/matrices. The distributed representation is learned based on their usage. This allows tokens used in similar ways to have similar representations, naturally capturing their relation. We have classified the embedding models into two broad categories:

##### Non-contextual

Non-contextual representation is short-ranged. It just converts each token into one vector without considering the context in which they are used. Two same tokens will have the same representation disregarding their different semantic meanings. Two common algorithms in this category are Word2vec and Glove. Here, Word2vec builds upon concepts from the Bag-of-Words model.

**Figure 5.**
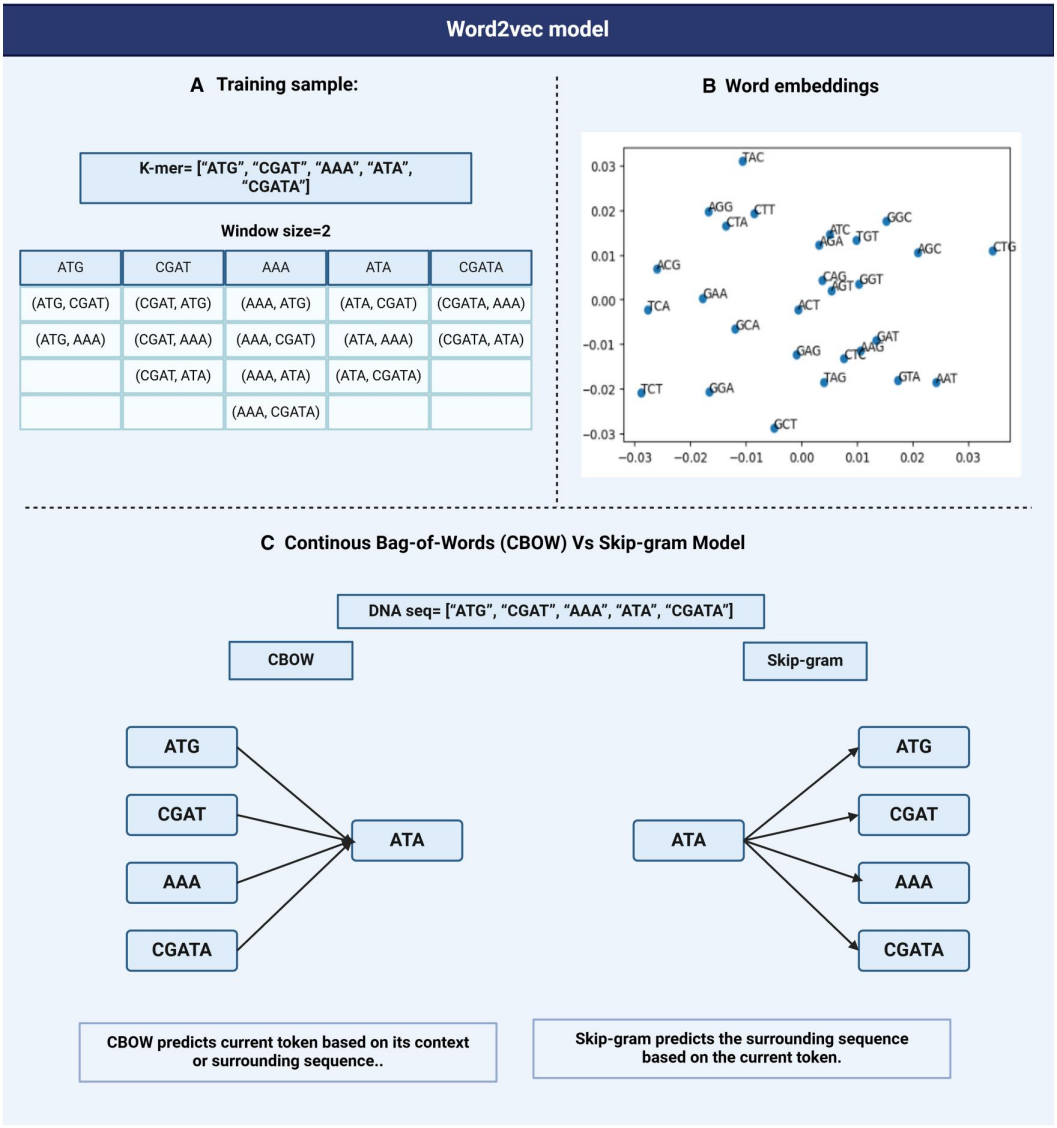
Illustration of Word2Vec model applied to k-mer sequences: (**A**) Training sample showcasing how Word2Vec processes a k-mer sequence using a window size of 2, traversing through the sequence to capture context-based relationships between neighboring k-mers. (**B**) A sample k-mer embedding plot: A graphical representation of the k-mer embeddings generated by the Word2Vec model. The X and Y axes indicate the relative distance between the k-mers, reflecting contextual similarities. Dense clusters suggest high similarity between k-mers, while loosely clustered or outlier k-mers suggest lower similarity or significant deviation from central groups. (**C**) This panel illustrates the difference between CBOW model and skip-gram model using a sample k-mer sequence.

- Bag-of-Words(BoW)
  BoW model [49] is a fundamental approach in NLP for representing text data by treating each document of the corpus as a collection or bag of words. BoW creates vocabulary from the unique tokens in the corpus. Each document is represented by a vector showing the frequency of the token(s) in each document. As a downside, this model ignores the order and grammar of the words losing contextual relationship. The large vocabularies created by BoW model results in computationally inefficient and sparse representations.
- Word2Vec
  Word2Vec [16] is a statistical method that enhances the BoW model by introducing learning dense word embeddings using neural networks. Word2Vec achieves this by implementing two strategies: Continuous Bag-of-Words, or CBOW model and Continuous Skip-Gram Model.
  CBOW is a representation of text that describes the occurrence of words within a document. This model is only concerned with whether known words occur in the document, not where in the document. We convert a corpus of text of variable-length (unstructured data) into a fixed-length vector which is structured and well-defined and thus preferred by NLP Algorithms. The CBOW model learns the embedding by predicting the current word based on its semantics or context. The continuous skip-gram model on the other hand, learns by predicting the surrounding words given a current word. Both models are focused on learning about words given their local usage context, where the context is defined by a predefined window of neighboring words. For Example, a window size of 2, means that for every word, we will consider the 2 words behind and the 2 words after it (Fig. 5).
  The Skip-Gram model works well with small-scale data, and better represents rare words or phrases. However, the CBOW model trains faster than Skip-Gram and better represents high-frequency words, thus giving a slightly better accuracy. The key benefit of the approach is that high-quality word embeddings can be learned efficiently i.e. with low space and time complexity, allowing larger embeddings to be learned from large-scale data. DNA2vec [50] is the DNA equivalent of Word2vec i.e. it is used specifically for applying the Word2vec approach to DNA sequences. It is used to transform tokens of variable length k-mers preferably 3–8 using the skip-gram model approach and thus captures efficient information of the sequences. Another example is the kmer2vec [51] model which is more focused on embedding fixed-length k-mers, typically between 3 and 6 to capture sequence relationships. This model is useful for tasks that require uniform k-mer lengths such as identifying specific patterns within genomic sequences. Models such as Gene2Vec are employed for various tasks like predicting N6-Methyladenosine (m6A) sites [52] and generating the representation of genes using gene co-expression patterns [3]. Similarly, Protein2vec is used for predicting protein-protein interaction [53], and aligning them across different species [54].
- Global Vectors for Word Representation (GloVe)
  The GloVe model [55] is an extension of the Word2vec model for efficiently learning word vectors. Word2Vec only captures the local context of words. GloVe considers the entire corpus and creates a large matrix from the words in the corpus. The matrix has the words as the rows and their occurrence frequency as the columns for a corpus of text. For large matrices, we factorize the matrix to create a smaller matrix. GloVe combines the advantages of two-word vector learning methods: matrix factorization and local context window methods like Skip-gram which reduces the computational cost of training the model. The resulting word embeddings are different and improved. Word tokens with similar semantic meanings or similar contexts are placed together, for example, “queen” and “women.” Being an extension to Word2vec, GloVe can be used for the representation of biological sequences as well. In a recent study, the GloVe model was integrated into a hybrid machine-learning model to classify gene mutations in cancer [56]. DeepMethylation, a methylation predictor uses GloVe embeddings to encode DNA sequences, performing better than the traditional models [57]. These embeddings are also used for RNA methylation site prediction [58].

##### Contextual

1. Embeddings from Language Models (ELMo)
  ELMo offers another way to convert a corpus of text into vectors or embeddings. ELMo is a bidirectional language (BiLM) model implemented using character-level convolutional neural network (CNN), two Bidirectional Long Short-Term Memory Network (BiLSTM) and task-specific layer. Each BiLSTM layer has 2 passes, a forward pass, and a backward pass. It first converts the words into vectors. These vectors act as inputs to the first layer of the BiLSTM model. The forward pass extracts and stores the information about the word in vector form and the context before it. The backward pass extracts and stores the information about the word and the context (other words) after it. This information contributes to the formation of intermediate word vectors which act as an input to the second layer of BiLSTM. The final representation is done by the weighted sum of the initial character-level CNN output and the of both BiLSTM layers. Recent studies use ELMo-based models to create embeddings for downstream tasks like protein structure prediction [59, 60]. ELMo embeddings are also used to improve the performance of NER models for identifying biomedical entities such as gene and protein names and cell lines [61].
2. Transformers
  Transformers introduced by [62] are a type of neural network architecture used for NLP tasks. Transformers make use of an attention mechanism i.e. when a transformer looks at a piece of data, like a DNA sequence, it does not just focus on one part. It pays attention to all the different parts at the same time. This enables transformers to capture complex relationships and dependencies within genomic sequences, identifying meaningful patterns across the data. A key component of transformer-based models is positional embeddings, which encode the order of tokens and help retain sequential information, regardless of the tokenization strategy used. A transformer model uses self-attention mechanisms (or its variants) to relate different positions within a single sequence. This allows each position in the sequence to attend to all other positions in the sequence, enabling the model to capture contextual information more effectively than traditional models.

##### BERT

BERT relies on this attention mechanism. In contrast to traditional (uni)directional models that read sequences from either the left or the right, transformers, including BERT use a bidirectional approach by using MLM in which tokens are masked at different intervals and models use preceding and succeeding tokens to predict the hidden tokens. BERT is an encoder-only transformer model. It takes input sequences and transforms them into fixed-size representations that capture important features. Word2vec or GloVe models generate a single-word embedding representation for each word in the vocabulary, whereas BERT will form a contextualized embedding that takes into account the context for each occurrence of a given word and will be different for each word according to the sentence. This enables BERT to provide more nuanced and accurate representations. This feature can be useful for tasks like classification and understanding genomic sequences that can have messages read in both directions and function via both short and long-range interactions. In genomics, DNABERT is a specialized adaptation of BERT for DNA sequences [63]. It is pre-trained on short sections of genomic data, learning patterns, and relationships. A newer version, DNABERT-2, uses BPE and other techniques to improve performance [29]. GenomicBERT uses unigram empirical tokenization and BERT attention mechanism to capture contextual relationships across large-scale biological datasets [34]. Other models like BioBERT, focusing on biomedical literature are leveraged to extract meaningful information from a vast biomedical text corpus [41, 64]. Similarly, ClinicalBERT is employed in medical decision-making by analyzing narrative data such as clinical notes [65].

##### Generative pre-trained transformers

Generative pre-trained transformers (GPT) are a type of transformer model but follow a different architecture than BERT. GPT models are based on a decoder-only framework. A decoder takes the representations generated from an encoder and uses these to generate predictions. GPT is trained using tasks like autoregressive language modeling (ALM), where the model sees a series of sequences and predicts the next sequence. It will be suitable for tasks involving sequence prediction and understanding directional sequences in genomics. Numerous models inspired by GPT such as DNAGPT [66], BioGPT [67], and GeneGPT [68] are being pre-trained on genomic sequences and biomedical literature whereas, scGPT [69] models single cell RNA expression profiles. The generalized models are then employed in various specialised tasks. For example, DNAGPT has been specifically designed for DNA sequence tasks such as mRNA abundance regression, and artificial genomes generation, while BioGPT focuses on generating and mining biomedical text.

### GLM enhances ML applications in genomics

ML has revolutionized genomics by providing powerful tools and methods for a range of applications [70, 71]. GLM further enhances the ML-driven genomics research by leveraging transformer-based architectures and DL techniques to model genomic sequences as a structured language. GLM holds promise to enhance the applications both in individual genomic sequences and integrative multi-omics or multimodal approaches.

#### Predictive modeling using classification and regression approaches

ML techniques are frequently used in classification tasks such as identifying gene expressions, categorizing cell types, protein classification, and distinguishing between healthy and diseased tissues. Early ML models primarily relied on handcrafted features derived from numerical encodings of genomic sequences, such as k-mer frequencies, one-hot encodings, and position weight matrices or numerical measurements of genic features. These methods, while effective for certain tasks like sequence classification and motif discovery, often struggled to capture long-range dependencies and complex genomic structures. Previous reviews [72–75] have highlighted the growing role of machine learning in genomics and precision medicine. They have discussed the increasing complexity and scale of biological data and applications of suitable AI-driven models for uncovering patterns and making predictions in genomics.

With the advent of deep learning, sequence-based models such as CNNs and recurrent neural networks (RNNs) improved feature extraction from raw genomic data. A detailed review discusses the prevalent neural network architecture and various DL resources to develop such methodologies [12]. However, these models were limited in handling the vast and intricate context within genomic sequences.

The application of LLMs for biological text [76] a paradigm shift by treating DNA, RNA, and protein sequences as a structured language [77]. Inspired by NLP, transformer-based architectures can now learn meaningful representations of genomic sequences without requiring extensive feature engineering. These models effectively capture the short- and long-range of the genome, enabling better predictions in gene expression, variant effect analysis, and multi-omics integration.

For example, sequence embedding techniques like Word2Vec [16] combined with Random Forest (RF) [78] and CNN [79] have been implemented. These approaches adopt GLM for feature engineering and representation learning. Similarly, CNN was used to identify N4-methylcytosine sites in DNA sequences, demonstrating the effectiveness of deep learning algorithms in genomic data analysis [2]. Different variation of Word2Vec-based models such as Bio2Vec and Prot2Vec were used to classify protein sequences. These embeddings helped capture the sequences’ semantic relationship, improving classification accuracy [1]. In another case, word embeddings combined with a deep learning framework were used to identify DNA replication origins [7]. Using a regression approach, a Residual Fully Connected Neural Network (RFCN) was employed for predicting gene regulatory networks [80]. In another study, LSTM and CNN models were used to predict RNA modifications using one-hot encoding and RGloVe embeddings, showcasing the versatility of neural network models in handling diverse genomic data types [58]. There is now a growing trend in developing foundational models for genomics (DNA, RNA, and proteins), which can be fine-tuned for specialized applications [29, 34, 81].

#### Sequence generation

GLMs, particularly transformer-based models like BERT and GPT are apt at generating new sequences of nucleotides or amino acids that conform to the grammar and structure of the genetic code. These models leverage NLP techniques to capture genomic data’s contextual relationships.

Genomic sequences exhibit a time series like property where nucleotides (A, C, G, T) or amino acids are organized into meaningful units whose meaning is dependent on their context. These units can range from k-mers and genes to larger genomic regions. GLMs are designed to capture this hierarchy, allowing for the generation of sequences that incorporate the genomic context.

GLMs consider the surrounding nucleotides or amino acids to accurately predict the next element in the sequence [82]. This contextual awareness is essential for generating biologically plausible sequences that align with the rules of the genetic code.

GLMs utilize various language modeling techniques to represent and generate nucleotide or amino acid sequences [83]. One prominent approach is ALM used by GPTs, which predicts the next token (nucleotide or amino acid) in a sequence based on the preceding tokens [84]. This technique allows GLMs to generate novel genomic sequences and aid in tasks such as sequence completion, mutation prediction, or identifying regulatory regions within genomes. These capabilities highlight the versatility and potential impact of applying language modeling to genomics.

#### Multi-modal data integration

The vast size of genomic data poses a significant challenge for its integration with other digital data types. Using GLM large genomic datasets can be effectively condensed into a set of tokens, matrices, and vectors. These tokens contain important features of the genomic data which are suitable for integration with other modalities of data. One example is the need of combining genetic information with the physiological markers routinely collected in the healthcare and stored in the electronic health record (EHR) systems. These condensed features reduce the storage requirement and at the same time enable efficient interpretation due to their high relevancy. Summarized genomic information such as genomic variants or relevant biomarkers have been successfully integrated with the EHR data to build joint representations [85, 86].

Similarly, once condensed, genomic features derived from sequence data can be seamlessly integrated with other *-omics* data (such as transcriptomics, proteomics, and metabolomics), which are captured through large-scale numerical profiling. This multimodal integration approach scales effectively across various matched modalities, regardless of the system under study.

#### Functional annotation

Functional annotation is an important process in genomics that involves identifying and assigning biological information to raw sequences and their products. This process helps in understanding the functional elements of a genome, such as genes or proteins, and their roles in biological processes and pathways.

Historically, tools like BLAST (Basic Local Alignment Search Tool) were used to perform sequence similarity searches to infer functional information based on the similarity of the unknown sequence to the known sequences. BLAST identifies regions of local similarity between sequences and aligns them to determine the likelihood of functional and evolutionary relationships. This approach, while effective, is limited by its dependency on existing annotations and its linear, non-contextual nature [87]. This method often needs to be revised when dealing with novel sequences or those with low similarity to known genes.

In the context of ML, functional annotation can be enhanced by both rule-based and DL approaches in an alignment-free manner [88]. Rule-based models are interpretable and can report features that are important for a predicted outcome. These models are built on predefined rules and statistical correlations, making it straightforward to understand which features contribute to a specific prediction. For instance, identifying DNA modification sites using rule-based algorithms can yield easily interpretable results, allowing researchers to directly correlate specific nucleotide patterns with functional annotations [2].

DL models, such as those based on transformers, offer a more complex but powerful approach to functional annotation. These models can automatically learn intricate patterns in genomic data, providing insights that might be missed by rule-based methods. For example, BERT and other transformer models can generate attention scores that highlight which parts of the input sequence are most influential for a given prediction. These attention scores can then be mapped back to biological functions, helping to assign biological relevance to important features. Similarly, RNA-FM [89] is a large-scale GLM foundation model based on BERT that was used for functional annotation by learning the sequential information from the unlabeled sequences. Another example is DeeRect-PromID [90], a model developed for human promoter annotation. It identifies the relevant patterns from promoter regions and helps in gene regulatory network prediction. In genomics, attention mechanisms within transformer models have been used to predict enhancers, promoters, and other regulatory elements by identifying key sequence motifs and their interactions [3]. Similarly, specialized approaches like DNABERT [82] and genomicBERT [36] focus on DNA sequences, offering more precise annotations by leveraging the bidirectional nature of BERT to capture context from both directions in the sequence.

NSP in BERT models enhances function annotation by understanding the relationship between genetic elements. In identifying operons or polycistronic messages in prokaryotic genomes, NSP can help predict whether a sequence of genes is likely to be transcribed together, revealing functional units within the genome. For instance, in a study of bacterial genomes, NSP was used to predict operon structures, significantly improving the accuracy of functional annotations compared to traditional methods. By analyzing the sequence context, NSP can identify co-regulating genes, providing insights into their collective biological functions [91].

Overall, combining rule-based and deep learning methods provides a robust framework for functional annotation, enhancing our ability to decipher complex genomic information.

## Future directions

The application of NLP techniques in genomics, particularly through GLM, offers significant advancements in identifying, understanding, and predicting biological functions by using the sequence information alone. This interdisciplinary approach leverages the strengths of NLP to process and interpret genomic sequences as natural language, enabling new possibilities for research and medical applications.

One of the most promising applications of GLM is in personalized medicine. By training models on combined healthcare and genomic data, researchers can identify genetic markers associated with physiological and lifestyle factors. This will enhance robust prediction of susceptibility to various diseases. For instance, deep learning models can analyze patterns in DNA sequences associated with genetic disorders, potentially leading to early diagnosis and personalized treatment plans. For instance, BERT-based models have shown great potential in identifying disease-related gene expressions and mutations, offering insights that can guide clinical decision-making [5].

GLM also plays a critical role in functional genomics, where understanding gene function and regulation is paramount. The ability to accurately annotate genes and regulatory elements helps in constructing detailed maps of genetic interactions and pathways [29, 92, 93]. These annotations are crucial for elucidating the mechanisms of gene expression and regulation, which can lead to the discovery of new therapeutic targets.

While the future of GLM in genomics is promising, there are significant limitations. Training on a variety of large-scale genomic datasets is computationally demanding, requiring substantial processing power and time. Like other ML approaches, class imbalance in genomic datasets also presents a challenge, as rare sequences might get overlooked, potentially biasing predictions. The lack of standardized benchmarks in new sequencing projects will pose a critical challenge for evaluating the outcomes of language models. Choosing appropriate tokenization and vocabulary sizes for language models are not straightforward either. The optimal vocabulary size depends on the application, this parameter defines the size of the embedding and output layers. Large vocabularies reduce sequence length but demand more memory, while smaller vocabularies increase computational complexity due to longer sequences. Previous benchmarking studies [25] are being used to tune vocabulary size parameters. Hybrid approaches, such as subword tokenization, strike a balance by efficiently handling both common and rare words. Understanding how language models generate predictions in genomics is often more challenging than in natural language tasks, as the complete “grammar” and “vocabulary” of the genome are still being uncovered. For this reason, the risk of model hallucination, where the model produces information that does not accurately reflect the underlying data, is especially concerning in genomics. Ongoing research focuses on developing neural network architectures with enhanced performance for better generalizability and efficient handling of long genomic sequences. Significant progress in LLMs for GLM has occurred over the past four years, but scaling these successful approaches in standard academic research settings with limited computing power remains a challenge, requiring further optimization.

With these limitations in mind, future research in GLM may focus on developing more efficient models for the processing of large-scale genomic data while ensuring interpretability and validation benchmarks. GLM offers enormous potential for understanding genetic information and improving genomic medicine research.

## Supplementary Material

bpaf022_Supplementary_Data
