# Supplementary material for: Genome language modeling (GLM): a beginner’s cheat sheet
Source: Biol Methods Protoc. 2025 Mar 25;10(1):bpaf022. doi: 10.1093/biomethods/bpaf022 (PMC12077296; doi:10.1093/biomethods/bpaf022)
Supplement: bpaf022_Supplementary_Data [file bpaf022_supplementary_data.zip › Table 4 Supplementary table.docx]

**Table 4** Supplementary table: Sequence processing methods

| **Method name** | **Method type** | **Tokenizer strategy** | **Language**  **restriction** | | **Reversibility** | **Application** | |
| --- | --- | --- | --- | --- | --- | --- | --- |
| Conventional word split-  ting | Rule-based tok-  enization | Separates words based on  space and/or punctuation. | Space-separated  languages | | True | Natural | Language |
| Conventional sentence  splitting | Rule-based tok-  enization | Separates sentences based  on full stops. | Full-stop separated  languages | | True | Natural | Language |
| Penn TreeBank | Rule-based tok-  enization | Separates contracted  words. | Use of contracted  words in the lan-  guage | | True | Natural | Language |
| TweetTokeniser | Rule-based tok-  enization | Separates audio streams  in the form of string into small tokens based on  space and/or punctuation. | Space-separated  languages | | False | Natural | Language |
| MWET (Multi-Word  Expression) | Rule-based tok-  enization | Processes tokenized set  and merges MWE into  single tokens. | Needs  tokens. | predefined | True | Natural | Language |
| TextBlob | Rule-based tok-  enization | Separates text into tokens  based on space, punctua-  tion, and/or tabs. | None | | True | Natural | Language |
| spaCy | Rule-based tok-  enization | Separates text(various  languages) into words  based on space. | Space-separated  languages | | True | Natural | Language |
| GenSim | Rule-based tok-  enization | Separates text based on  space and/or punctuation. | Contracted words | | True | Natural | Language |
| Keras tokenizer | Rule-based tok-  enization | Separates text into integer  sequence or vector that has a coefficient for each  token | None | | False | Natural | Language |
| Moses | Rule-based tok-  enization | Separates text based on  spaces. | Space-separated  languages | | True | Natural | Language |
| MeCab | Sequence segmen-  tation | Segments sentences into  their parts of speech. | None | | False | Natural | Language |
| KyTea | Sequence segmen-  tation | Segments sentences into  their parts of speech and  pronunciation tags. | None | | False | Natural | Language |
| Byte Pair Encoding  (BPE) | Sequence segmen-  tation (requires pre-tokenized  input) | Recodes sequences into a  standardized format by frequency. | None | | False | Natural | Language |
| Wordpiece | Sequence segmen-  tation (requires pre-tokenized  input) | Recodes sequences into a  standardized format by likelihood. Variant of BPE | None | | False | Natural | Language |
| Unigram | Sequence segmen-  tation (requires pre-tokenized input) | Recodes sequences into a  standardized format by likelihood, producing a set of tokens and their proba-  bilities. Variant of BPE | None | | False | Natural | Language |
| SentencePiece | Data-driven tok-  enization | Empirically derives tokens  by sequence segmentation with BPE or its variants  i.e. WordPiece, Unigram. | None | | True | Natural | Language |
| k-mers | Rule-based tok-  enization | Sequence data is split into  tokens of fixed length | None | | False | Biology | |
| Khmer | Rule-based tok-  enization | Sequences are arbitrarily  split into subsequences. | None | | False | Biology | |
| Tab | Rule-based tok-  enization | Separates text based on  tabs between them. | Tab-separated text | | True | Natural | Language |
